# Non-equilibrium synergistic effects in atmospheric pressure plasmas

**DOI:** 10.1038/s41598-018-22911-8

**Published:** 2018-03-19

**Authors:** Heng Guo, Xiao-Ning Zhang, Jian Chen, He-Ping Li, Kostya (Ken) Ostrikov

**Affiliations:** 1 Department of Engineering Physics, Tsinghua University, Beijing, 100084 P. R. China; 2 Laboratory for Space Environment and Physical Sciences, Harbin Institute of Technology, Harbin, 150001 P. R. China; 3 School of Chemistry, Physics and Mechanical Engineering, Queensland University of Technology, Brisbane, Queensland 4000 Australia; 4 CSIRO-QUT Joint Sustainable Processes and Devices Laboratory, P. O. Box 218, Lindfield, NSW 2070 Australia

## Abstract

Non-equilibrium is one of the important features of an atmospheric gas discharge plasma. It involves complicated physical-chemical processes and plays a key role in various actual plasma processing. In this report, a novel complete non-equilibrium model is developed to reveal the non-equilibrium synergistic effects for the atmospheric-pressure low-temperature plasmas (AP-LTPs). It combines a thermal-chemical non-equilibrium fluid model for the quasi-neutral plasma region and a simplified sheath model for the electrode sheath region. The free-burning argon arc is selected as a model system because both the electrical-thermal-chemical equilibrium and non-equilibrium regions are involved simultaneously in this arc plasma system. The modeling results indicate for the first time that it is the strong and synergistic interactions among the mass, momentum and energy transfer processes that determine the self-consistent non-equilibrium characteristics of the AP-LTPs. An energy transfer process related to the non-uniform spatial distributions of the electron-to-heavy-particle temperature ratio has also been discovered for the first time. It has a significant influence for self-consistently predicting the transition region between the “hot” and “cold” equilibrium regions of an AP-LTP system. The modeling results would provide an instructive guidance for predicting and possibly controlling the non-equilibrium particle-energy transportation process in various AP-LTPs in future.

## Introduction

Atmospheric-pressure low-temperature plasma (AP-LTP) operates in a non-equilibrium state under most operating conditions. One of the major features of AP-LTPs is that the characteristic electron energy ranges from a few eV to 10 eV, while the heavy-particle temperature varies from around the room temperature to a level which is comparable to but usually lower than the electron temperature^1,2^. This results from the physical facts that the significant mass difference between electrons and heavy particles (e.g., molecules, atoms and their ions), and consequently, the lower energy exchange rate between the subsystems of electrons and heavy particles compared with that of power input; and the inevitable involvement of the solid walls in the production and application of the laboratory plasmas. There may exist three types of non-equilibria in an AP-LTP system: (i) Non-electrical-equilibrium (NEQ): if a conductive or non-conductive object is inserted into the plasma region, a sheath deviating from the quasi-charge neutrality condition arises between the object surface and the plasma region^3,4^, and most of the total electric potential drop occurs at this NEQ region. (ii) Non-local-thermodynamic-equilibrium (NLTE): the low mass ratio of electrons to heavy particles in plasmas (e.g., 10^−5^ for argon) leads to an insufficient elastic energy exchange between electrons and heavy particles; and thus, the whole plasma system will be in an NLTE state, especially if the plasma density is also not high enough. (iii) Non-local-chemical-equilibrium (NLCE): since the mean electron temperature is around several electron volts (eV), which is lower than the inelastic collision energy thresholds of tens of eV, the excitation and ionization rates decrease significantly particularly when interacting with cold gas; and thus, the plasma will be in an NLCE state.

The non-equilibrium of the AP-LTPs is of great importance because we can re-distribute the input energy to other types of energies at various freedoms for their wide applications, e.g., kinetic energy in plasma flow control^5^, electronically and vibrationally excited energies in plasma assisted combustion^6^, energies stored in various reactive species with optimized non-chemical equilibrium spatial distributions in plasma-aided synthesis of nano-scale materials^7–12^, etc. However, it is so difficult to describe, explain, and use such non-equilibria in the AP-LTPs since three types of non-equilibria usually appear in the same discharge. Even worse, there may co-exist the equilibrium and three types of non-equilibria in the same AP-LTP system, for example, the free-burning arc with a higher arc current. Therefore, it is rather difficult to describe this strongly coupled phenomenon self-consistently. In our opinion, except for the NEQ effect in plasma sheath^13–15^, the NLCE process plays an important role among these three types of non-equilibria for various types of AP-LTPs with wide applications such as plasma biomedicine, advanced materials processing, plasma-assisted ignition or combustion, environmental protection, etc.^1,2,16,17^. This is because the chemical reactions determine not only the spatial distributions of the species number densities but also the corresponding energy transfer processes significantly. For example, in an LCE-NLTE modeling of an arc discharge by Haidar^18^, a physically incorrect increase in the elastic collision frequency between electrons and heavy particles had to be made for obtaining the close electron and heavy-particle temperature distributions in the arc fringe region. This is because the approaching of the electron and heavy-particle temperatures to each other in the arc fringe region results physically from the energy re-distribution between the subsystems of electrons and heavy species due to the chemical reactions. In recent years, although some modeling work on the NLCE plasmas have been conducted, for example, the work by Tanaka^19^ and Baeva *et al*.^20,21^, the mutual interactions between the non-equilibrium effects of NLCE and NLTE have not been completely revealed with no considerations of the coupled energy and particle balance processes in the same plasma system. Furthermore, even purely for the NLTE situations, the spatially non-uniform temperature distributions of the electron and heavy-particle subsystems would: (i) influence the energy transfer process of the electron or heavy-particle subsystem itself through the traditional heat conduction, convection processes, and the elastic collision process between electrons and heavy particles, ${Q}_{{\rm{eh}}}^{{\rm{el}}}$; (ii) result in an extra energy transfer process to the electron or heavy-particle subsystem due to the spatial gradient of the temperature ratio, the term containing $\overrightarrow{\nabla }\,\mathrm{ln}\,\theta$ (here *θ* = *T*_e_/*T*_h_) in the energy conservation equations of electrons and heavy particles as shown in refs^22,23^. To our knowledge, the second non-equilibrium energy transfer process has never been reported up to now. This, to some extent, indicates a mutual influence between the electron and heavy-particle subsystems.

**Figure 1:**
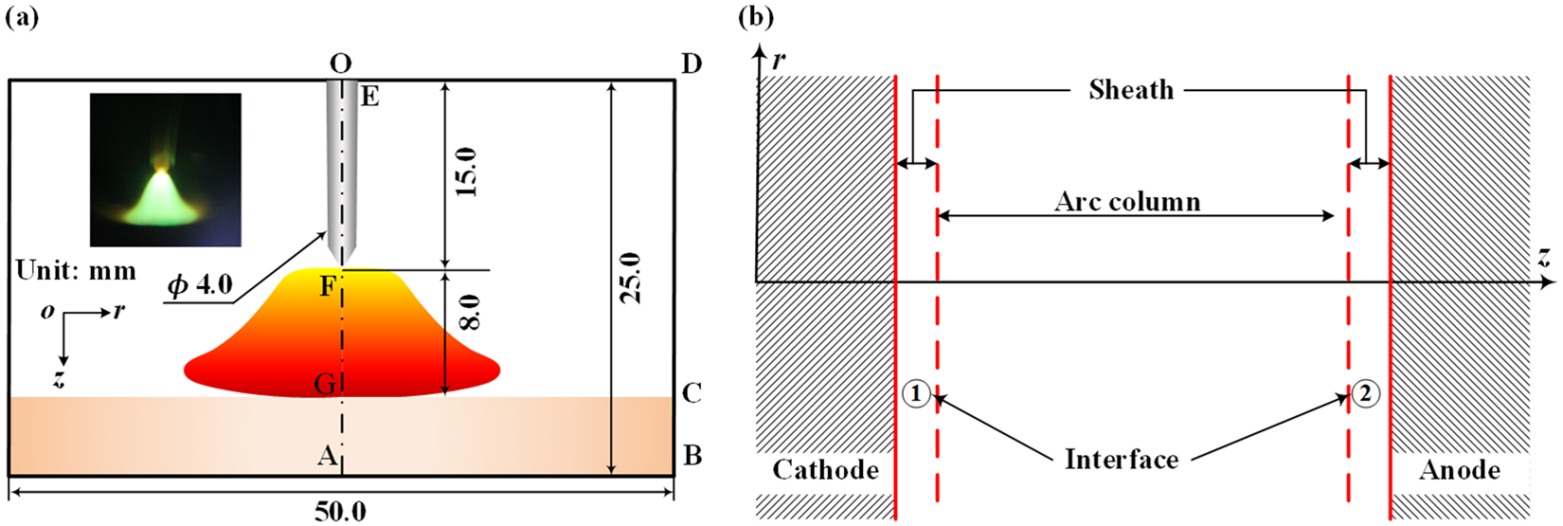
Schematic of an atmospheric free-burning arc plasma **(a**) and the selected calculation domain used in this study (**b**).

In this study, an atmospheric free-burning argon arc plasma, as illustrated in Fig. 1(a), is selected as a typical AP-LTP. The goal of this report is to reveal the NLTE-NLCE synergistic effects, especially the self-consistent transition between the thermal-chemical equilibrium and non-equilibrium states in the same AP-LTP system based on the self-consistent considerations of the energy and particle balance processes in plasmas^23,24^. This result provides a deep understanding to and a complete evaluation of the strongly coupled thermal and chemical non-equilibria, and a constructive guidance for the possible control of the non-equilibrium particle-energy transportation process in the AP-LTPs in future.

## Modeling Results

In this study, three dimensionless parameters are employed to describe the non-equilibrium features of the plasmas: (i) the temperature ratio, *θ* (=*T*_e_/*T*_h_), to quantitatively describe the NLTE state of the plasmas; (ii) the chemical reaction ratio, *ξ* (=*r*_ion_/*r*_rec_), and the electron number density ratio, $\zeta (={n}_{{\rm{e}}}/{n}_{{\rm{e}}}^{\ast })$, to indicate the NLCE state of the plasmas, where *r*_ion_ and *r*_rec_ are the ionization and recombination rates of the chemical reaction Ar + e ↔ Ar^+^  + 2e, *n*_e_ and ${n}_{{\rm{e}}}^{\ast }$ represent the electron number densities based on the solution of the non-equilibrium governing equations and the Saha equation^25^ with an assumption of LCE, respectively. Therefore, the physical meanings of the preceding dimensionless parameters can be explained as: *θ* = 1.0 for the LTE state, while *θ* > 1 indicating the deviations of plasmas from the LTE state and a higher value of *θ* representing a higher non-thermal-equilibrium degree; similarly, *ξ* = *ζ* = 1.0 for the LCE state with a detailed balance of chemical reactions, and the higher or lower values than 1.0 for *ξ* and *ζ* indicating the higher non-chemical-equilibrium degrees.

For an atmospheric gas discharge plasma, frequent collisions between diverse particles are essential to sustain the plasma itself. The elastic collisions transfer energy and momentum between species. The inelastic collisions transfer not only energy and momentum but also charges through the processes of charge exchange, ionization, and recombination. Both of the elastic and inelastic collisions drive the plasma system to be in an equilibrium state. From the aspect of energy transfer, each subsystem (electrons or heavy particles) obtains energy through the Joule heating process from the external power supply ($e{\vec{J}}_{{\rm{j}}}\cdot ;\vec{E},\,{\rm{j}}={\rm{e}},{\rm{i}}$), and loses the energy to the surroundings via radiation, conduction and convection processes. Simultaneously, there exist the energy exchange processes between the electron and heavy-particle subsystems through the local elastic and inelastic collision processes (${Q}_{{\rm{e}}{\rm{h}}}^{{\rm{e}}{\rm{l}}}+{Q}_{{\rm{j}}}^{{\rm{i}}{\rm{n}}{\rm{e}}{\rm{l}}},\,{\rm{j}}={\rm{e}},{\rm{i}}$), and a non-local energy transfer process related to the spatial gradient of the temperature ratio (${\lambda }_{{\rm{j}}}^{{\rm{\theta }}}{T}_{{\rm{e}}}\overrightarrow{\nabla }\,\mathrm{ln}\,\theta$, where j = e and i, ${\lambda }_{{\rm{j}}}^{{\rm{\theta }}}$ represents the non-equilibrium thermal conductivity of species j^24^) which represents a newly-revealed energy transfer process and will be discussed in the following section.

**Figure 2:**
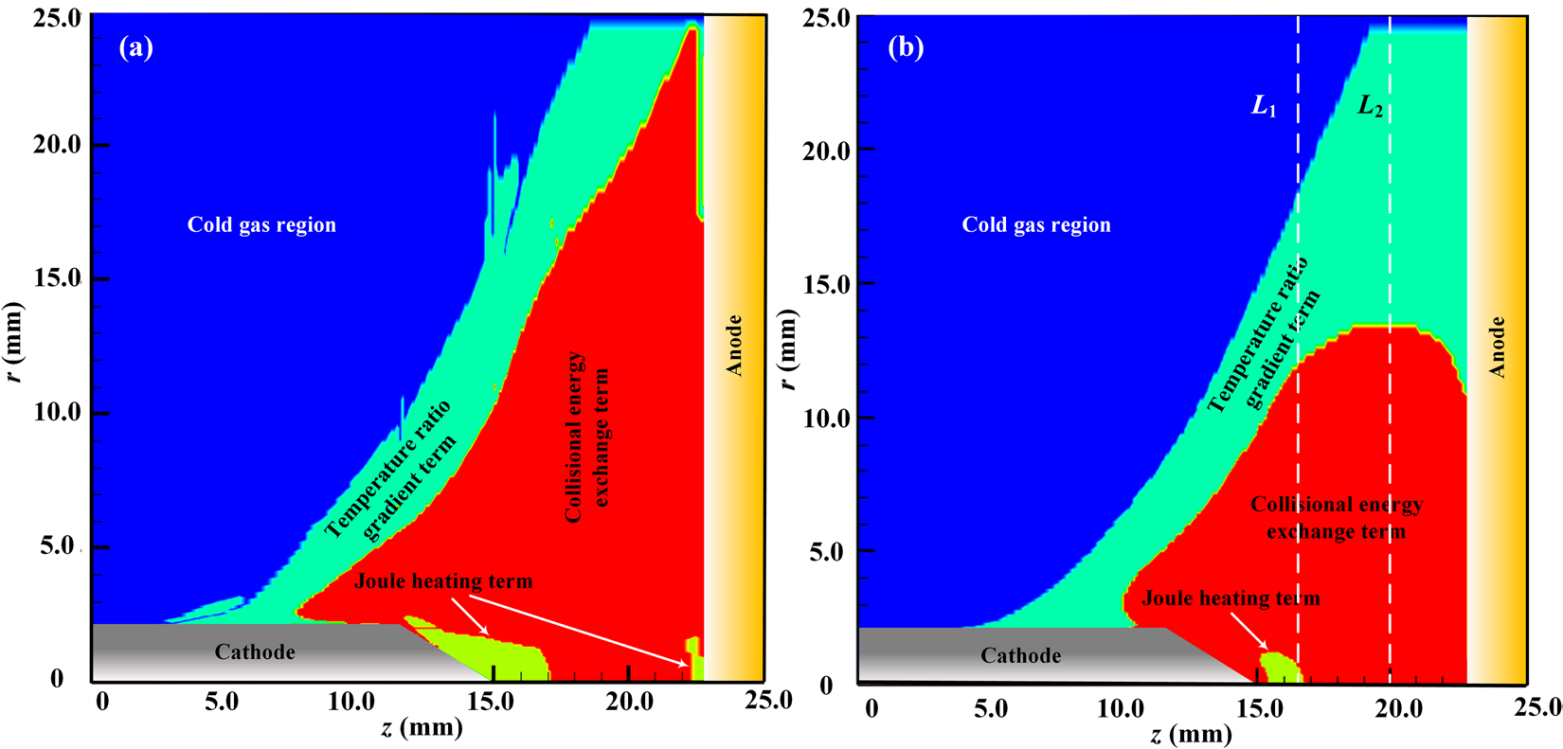
Illustrations of different dominated energy transfer processes in the arc column region for the electron (**a**) and the heavy particle (**b**) subsystems at *I* = 100 A. The two dashed lines in **(b**) indicate the two axial positions at the downstream of the cathode tip, i.e., *z* = 16.5 (*L*_1_) and 20.0 mm (*L*_2_), for presenting the radial profiles of the plasma parameters in Fig. 3.

**Figure 3:**
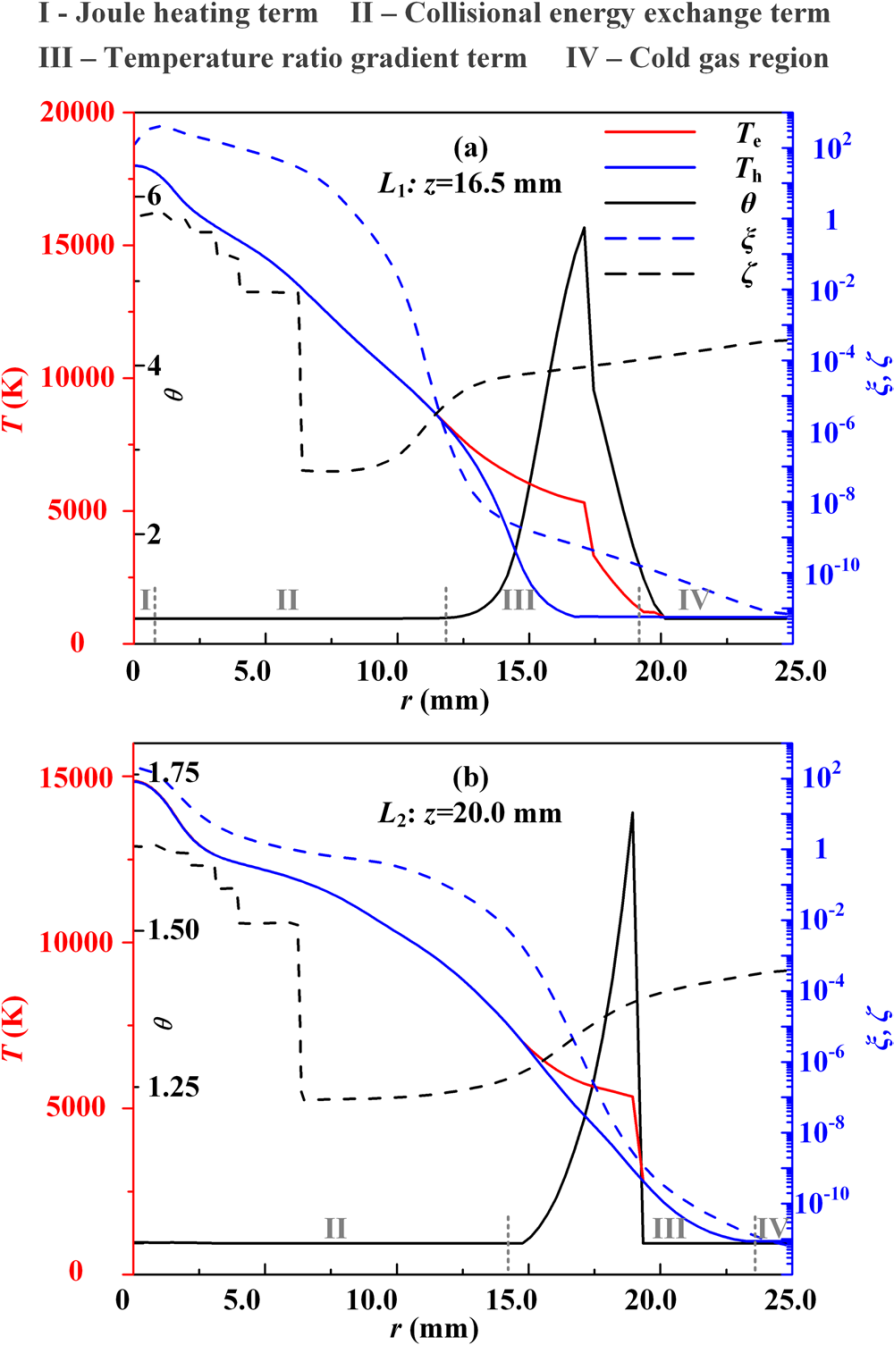
Radial profiles of the plasma parameters *T*_e_, *T*_h_, *θ*, *ξ* and *ζ* at the axial positions of *L*_1_: *z* = 16.5 mm (**a**) and *L*_2_: *z* = 20.0 mm (**b**) for the case of *I* = 100 A. The symbols I~III indicate the regions corresponding to the dominated energy transfer processes of Joule heating, collisional energy exchange and the term related to the spatial gradient of the temperature ratio for the heavy-particle subsystem in Fig. 2(b); while the symbol IV indicates the cold gas region.

Figure 2 illustrates the spatial distributions of the Joule heating energy deposition process and the two energy exchange processes between the electrons and heavy particles at an arc current of *I* = 100 A, where the regions with different colors represent the dominated energy transfer process among the preceding three ones and are designated as the “Joule heating term”, “Collisional energy exchange term” and “Temperature ratio gradient term”, respectively. The radial profiles of the plasma parameters, including *T*_e_, *T*_h_, *θ*, *ξ* and *ζ*, at 1.5 (*L*_1_) and 5.0 mm (*L*_2_) downstream of the cathode tip are shown in Fig. 3. It is seen clearly from Figs 2 and 3 that: (i) The Joule heating process rules the energy deposition process near the cathode tip and the center of the anode surface (for electrons) due to the higher current densities in these regions; and the Joule heating dominating region (Region I in Fig. 3) is larger in the electron subsystem than that in the heavy-particle subsystem because the current is mainly transported by electrons instead of ions. (ii) Due to the strong Joule heating effect, the gas is heated and partially ionized with a rapid expansion, and thus, experiences a collision-dominated process in both the Joule heating dominated region and the collisional energy exchange dominated region (Region II in Fig. 3). Just resulting from the frequent collisions between the electrons and heavy particles, the energy is exchanged sufficiently between these two subsystems, and *θ* stays nearly unity forming an LTE state. (iii) With a further expansion of the arc plasma, when the plasma meets the surrounding cold gas, a steep variation of *θ* occurs resulting from the violent interactions between the arc plasma and the cold gas; and thus, the steep decreases of the electron (*T*_e_) and heavy-particle temperatures (*T*_h_) occur in Region III of Fig. 3. The resulted large gradients of *T*_e_, *T*_h_ and *θ* play a significant role in the energy transfer process which will be discussed in detail in the next section. In addition, it should be emphasized that: (i) Although the radiation loss is not dominated at any spatial positions in the arc column region, it still plays a vital role in the energy transfer process which will also be explained below. (ii) In the blue region of Fig. 2, the electron number density decreases to a very small value with no current passing through, and thus, the preceding energy transfer processes of Joule heating, elastic and inelastic collisions and the energy transfer related to the temperature ratio gradient all become very weak. This means that no plasma exists and just a cold gas left, which is designated as Region IV in Fig. 3.

Figure 3 also shows the radial distributions of the chemical reaction ratio (*ξ*) and the electron number density ratio (*ζ*). It is seen that *ξ* reaches the maximum in the vicinity of the arc axis and becomes minimum approaching the arc fringe. In the region near the geometrical axis of the arc, owing to the higher current density near the cathode tip, there exist strong Joule heating and very frequent energy exchanges between electrons and heavy particles; thus, the ionization process is dominated with LTE and LCE features, i.e., *θ* ~ 1 and *ξ* ~ 1 in Region I. With approaching the arc fringe in the radial direction, both *T*_e_ and *T*_h_ decrease, and the decreasing rate of *T*_h_ is much faster than that of *T*_e_. This makes the value of *θ* increases to a maximum value first around *r* = 16.0 mm, and then, decreases to unity at the arc fringe where the cold gas dominated in Region IV. Correspondingly, the value of *ζ* experiences a drop to a minimum value and then a slight increase. The value of *ζ* is typically below unity with *r* > 5.0 mm. It means that the plasma departs from Saha equilibrium much more significantly than that of thermal equilibrium.

From the modeling results presented in Figs 2 and 3, it is seen clearly that the collision processes play a key role in the determination of the non-equilibrium characteristics of the arc plasmas. In the core region close to the arc axis, sufficient collisions between electrons and heavy particles result in an LTE-LCE state with a high electron number density and the equal values of *T*_e_ and *T*_h_. While with the violent interactions between the arc plasma and the cold surrounding gas, a strong quenching of plasma occurs with a significant recombination process (i.e., *ξ* ≪ 1 in Region III of Fig. 3) and an accompanied significant decrease of the electron number density. This process results in the insufficient elastic collision energy exchanges leading to a large discrepancy between the temperatures of electrons and heavy particles (i.e., *θ* ≫ 1). At the same time, the energy release during the recombination process also leads to a big energy loss of the electron subsystem, and thus, results in the equal temperatures of electrons and heavy particles when the plasma decays to an ordinary cold gas in Region IV of Fig. 3. We can see that a self-consistent transition from the LTE-LCE plasma state, which can be called as a “hot” equilibrium state, to an obvious NLTE-NLCE plasma state, and then, to an ordinary cold gas state, which can be called as a “cold” equilibrium state, is revealed based on this newly-developed complete non-equilibrium plasma model with no non-physical treatments to the electron-heavy-particle collisions^18^. In our discussions hereafter, we also call Region III as the “transition region” to emphasize its unique features physically.

## Discussions

The novel self-consistent physical-mathematical model^23,24,26^ is presented in the Methods and Validation Section. It is employed in this report for investigating the NLTE-NLCE synergistic effects in an AP-LTP system. For a non-equilibrium plasma system including electrons, ions and atoms as discussed in this study, starting from the Boltzmann equation for the distribution function of species j (*f*_j_) as

1 $$\frac{{\rm{D}}{f}_{{\rm{j}}}}{{\rm{D}}t}=\sum _{k=1}^{N}\iint ({f}_{{\rm{j}}}^{\prime}{f}_{{\rm{k}}}^{\prime} -{f}_{{\rm{j}}}{f}_{{\rm{k}}})g{\sigma }_{{\rm{j}}{\rm{k}}}{\rm{d}}{\rm{\Omega }}{\rm{d}}{\mathop{c}\limits^{\rightharpoonup }}_{{\rm{k}}},$$

we can obtain the following equations for the electrons (j = 1) and heavy particles (j ≥ 2), respectively, based on a modified Chapman-Enskog method^24^.

2 $$\frac{{\rm{D}}{f}_{1}^{(0)}}{{\rm{D}}t}=\iint {f}_{1}^{(0)}{f}^{(0)}({\varphi }_{1}^{\prime} +\varphi ^{\prime} -{\varphi }_{1}-\varphi )g{\sigma }_{11}{\rm{d}}{\rm{\Omega }}\,{\rm{d}}\vec{c}+\sum _{k=2}^{N}\iint {f}_{1}^{(0)}{f}_{{\rm{k}}}^{(0)}({\varphi }_{1}\text{'}-{\varphi }_{1})g{\sigma }_{1{\rm{k}}}{\rm{d}}{\rm{\Omega }}\,{\rm{d}}{\vec{c}}_{{\rm{k}}},$$

3 $$\frac{{\rm{D}}{f}_{{\rm{j}}}^{(0)}}{{\rm{D}}t}=\iint {f}_{{\rm{j}}}^{(0)}{f}_{1}^{(0)}({\varphi }_{1}^{\prime} -{\varphi }_{1})g{\sigma }_{{\rm{j}}1}{\rm{d}}{\rm{\Omega }}\,{\rm{d}}{\vec{c}}_{1}+\sum _{k=2}^{N}\iint {f}_{{\rm{j}}}^{(0)}{f}_{{\rm{k}}}^{(0)}({\varphi }_{{\rm{j}}}^{\prime} +{\varphi }_{{\rm{k}}}^{\prime} -{\varphi }_{{\rm{j}}}-{\varphi }_{{\rm{k}}})g{\sigma }_{{\rm{j}}{\rm{k}}}{\rm{d}}{\rm{\Omega }}\,{\rm{d}}{\vec{c}}_{{\rm{k}}},$$

with expanding *f*_j_ to its first order approximation, ${f}_{{\rm{j}}}={f}_{{\rm{j}}}^{(0)}(1+{\varphi }_{{\rm{j}}})$, under the assumption of ${\varphi }_{{\rm{j}}}\ll 1$; while the perturbation functions for electrons (${\varphi }_{1}$) and heavy species (${\varphi }_{{\rm{j}}},{\rm{j}}\ge 2$) can be expressed as

4 $${\varphi }_{1}=-{\overrightarrow{A}}_{1}\cdot \overrightarrow{\nabla }\,\mathrm{ln}\,{T}_{{\rm{h}}}-{\overleftrightarrow{B}}_{1}:\overrightarrow{\nabla }{\overrightarrow{v}}_{0}+\sum _{k=1}^{N}{\overrightarrow{C}}_{1}^{{\rm{k}}}\cdot {\overrightarrow{d}}_{{\rm{k}}}+{D}_{1}{Q}_{1}^{(0)}+\sum _{k=1}^{N}{\overrightarrow{E}}_{1}^{{\rm{k}}}{\omega }_{{\rm{k}}}\cdot \overrightarrow{\nabla }\,\mathrm{ln}\,\theta -{\overrightarrow{F}}_{1}\cdot \overrightarrow{\nabla }\,\mathrm{ln}\,\theta ,$$

5 $${\varphi }_{{\rm{j}}}=-{\overrightarrow{A}}_{{\rm{j}}}\cdot \overrightarrow{\nabla }\,\mathrm{ln}\,{T}_{{\rm{h}}}-{\overleftrightarrow{B}}_{{\rm{j}}}:\overrightarrow{\nabla }{\overrightarrow{v}}_{0}+\sum _{k=1}^{N}{\overrightarrow{C}}_{{\rm{j}}}^{{\rm{k}}}\cdot {\overrightarrow{d}}_{{\rm{k}}}+{D}_{{\rm{j}}}\,{Q}_{1}^{(0)}+\sum _{k=1}^{N}{\overrightarrow{E}}_{{\rm{j}}}^{{\rm{k}}}{\omega }_{{\rm{k}}}\cdot \overrightarrow{\nabla }\,\mathrm{ln}\,\theta -{\overrightarrow{F}}_{{\rm{j}}}\cdot \overrightarrow{\nabla }\,\mathrm{ln}\,\theta ,$$

with the corresponding diffusion driving forces

6 $${\overrightarrow{d}}_{1}=\frac{{\rho }_{1}}{\rho }\sum _{k=1}^{N}{n}_{{\rm{k}}}{\overrightarrow{X}}_{{\rm{k}}}-{n}_{1}{\overrightarrow{X}}_{1}+(\frac{{x}_{1}\theta }{D}-\frac{{\rho }_{1}}{\rho })\overrightarrow{\nabla }p+\frac{\theta p}{{D}^{2}}\overrightarrow{\nabla }{x}_{1},$$

7 $${\overrightarrow{d}}_{{\rm{j}}}=\frac{{\rho }_{{\rm{j}}}}{\rho }\sum _{k=1}^{N}{n}_{{\rm{k}}}{\overrightarrow{X}}_{{\rm{k}}}-{n}_{{\rm{j}}}{\overrightarrow{X}}_{{\rm{j}}}+(\frac{{x}_{{\rm{j}}}}{D}-\frac{{\rho }_{{\rm{j}}}}{\rho })\overrightarrow{\nabla }p+\frac{p}{D}\overrightarrow{\nabla }{x}_{{\rm{j}}}-\frac{{x}_{{\rm{j}}}(\theta -1)p}{{D}^{2}}\overrightarrow{\nabla }{x}_{1}.$$

**Figure 4:**
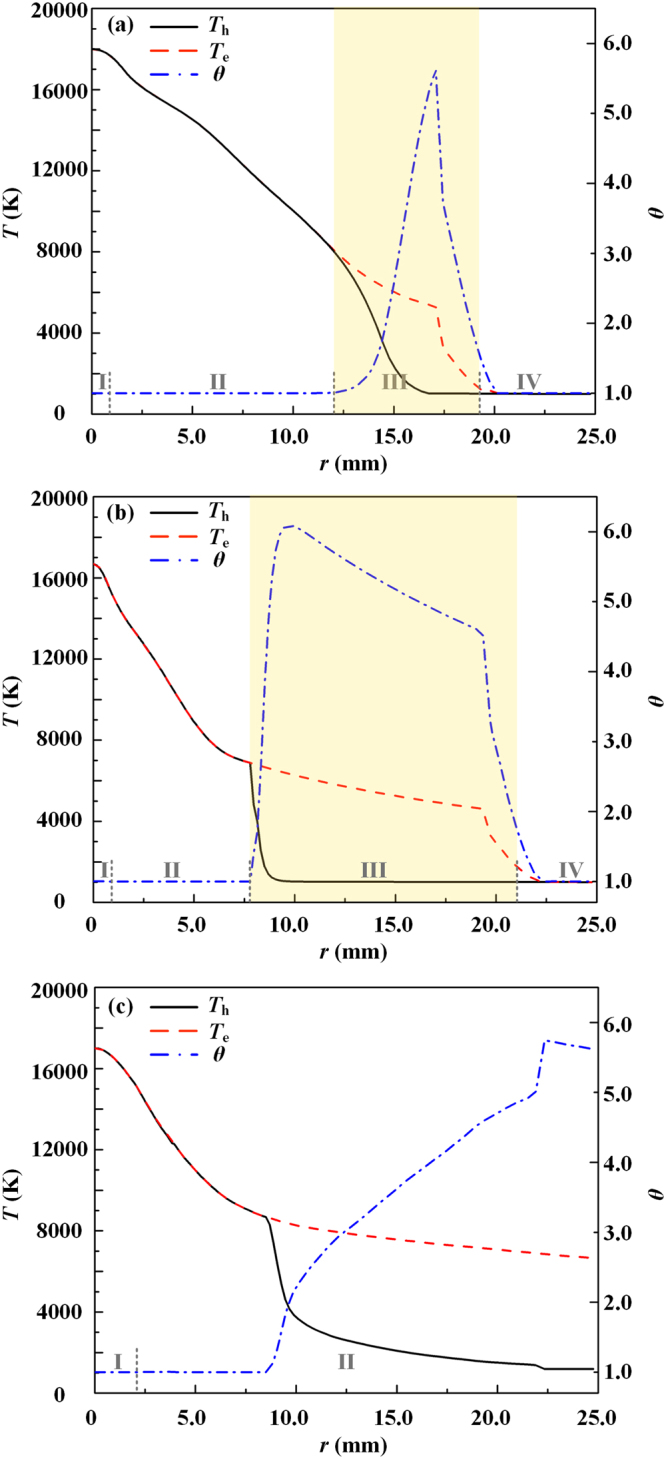
Comparisons of the calculated radial profiles of *T*_h_, *T*_e_ and *θ* at the axial location of *z* = 16.5 mm using different models for the case of *I* = 100 A. (**a**) Complete non-equilibrium model; (**b**) Non-equilibrium model with no terms containing $\vec{{\rm{\nabla }}}\,{\rm{l}}{\rm{n}}\,\theta$ in Equations (10) and (11); and (**c**) 2-T model. The corresponding various dominated energy transfer regions as those in Figs 2 and 3 are also indicated in this figure and the transition region is highlighted with a light-yellow background in (**a**) and (**b**).

The meaning of the symbols appearing in the preceding equations can be referred to ref.^24^. The major features of the non-equilibrium plasma model employed in this report^23,24^ can be briefly summarized as follows:

Firstly, the major difference between the species Boltzmann equations in this study [Equations (2) and (3)] and those employed in previous studies^27,28^ is that both the physical fact *m*_e_/*m*_h_ ≪ 1 and the inclusion of the coupling between the electron and heavy-particle subsystems are considered. This leads to the appearance of the term $\iint {f}_{{\rm{j}}}^{(0)}{f}_{1}^{(0)}({\varphi }_{1}^{\text{'}}-{\varphi }_{1})g{\sigma }_{{\rm{j}}1}{\rm{d}}{\rm{\Omega }}\,{\rm{d}}{\overrightarrow{c}}_{1}$ in Equation (3) and the term $\sum _{k=1}^{N}{\overrightarrow{C}}_{{\rm{j}}}^{{\rm{k}}}\cdot {\overrightarrow{d}}_{{\rm{k}}}$ in Equations (4) and (5) representing the interactions between the electrons and heavy particles. Therefore, a complete set of diffusion coefficients can be obtained which ensures the exact mass conservation in a plasma system and differs from the simplified theory presented in refs^27,28^. The diffusion between electrons and heavy species represents the mass exchange process between these two subsystems; and there exists a significant influence on the diffusion coefficients due to the interactions between electrons and heavy particles. For example, the relative discrepancies between the thermal diffusion coefficients of heavy particles (i.e., argon atoms and ions) with or without considering the electron-heavy-particle interactions can reach 58% for *θ* = 3 at *T*_e_ = 14000 K^26^. Furthermore, an energy exchange between the subsystems of electrons and heavy particles must be involved during the species diffusion process. Under an NLCE condition, this energy exchange process is represented by the terms ${Q}_{{\rm{e}}}^{{\rm{inel}}}$ and ${Q}_{{\rm{h}}}^{{\rm{inel}}}$ in the electron and heavy-particle energy conservation equations [Equations (10) and (11)]. Just this diffusion-related inelastic energy exchange process determines dominantly the self-consistent spatial temperature variations of the electrons and heavy-particles, i.e., the transition region (Region III in Fig. 3). We can explain physically the radial temperature variations in Fig. 3 as follows: Starting from the arc axis, when the plasma expands towards the arc fringe, both the electron and heavy-particle temperatures decrease due to a lower current density and lower Joule heating. This also leads to lower species number densities, and consequently, lower collision frequencies between electrons and heavy particles. Thus, an NLTE plasma exits with a significant increase of *θ*, which has also been revealed in the recent work by Baeva *et al*.^20^. While with a further decrease of *T*_e_ around lower than 5000 K, the recombination rate increases and exceeds the ionization rate significantly. Under such conditions, the inelastic collision energy exchange process plays a dominant role in controlling the energy re-distribution between the subsystems of electrons and heavy particles, and thus, makes the values of *T*_e_ and *T*_h_ close to each other. This means that the value of *θ* becomes unity again in the arc fringe region where the plasma decays to an ordinary cold gas quickly due to the strong interactions between the plasma mainstream and the cold surrounding gas. Our numerical modeling shows that in the transition region with a larger value and gradient of *θ* in Fig. 3, the radiation loss of electrons can be neglected due to the lower value of *T*_e_ (<7500 K in Fig. 3); and simultaneously, the Joule heating term is also very small because of the negligible current density in this region. Thus, the energy loss of electrons mainly comes from the electron-heavy-particle elastic collisions with a lower efficiency; while in contrast, the energy exchange between the heavy particles and the surrounding cold gas is sufficiently high, which leads to a much faster decrease of the heavy-particle temperature than that of electrons within the radial region of 13~16 mm as indicated in Fig. 4. However, with further approaching the arc fringe in the radial direction, the fast electron decaying due to the recombination with ions takes away much more energy from the electron subsystem, which leads to a steep drop of the electron temperature in the radial direction from 16~20 mm, and a fast decrease of *θ* to unity in the cold gas region. For validating the important role of the chemical reactions on this self-consistent variation of the species temperatures, the modeling results based on the two-temperature (2-T) model are presented in Fig. 4. It can be seen clearly that the electron temperature is always much higher than that of heavy particles along the radial direction away from the arc axis, even though in the cold gas region; and thus, the transition region cannot be predicted physically.

**Figure 5:**
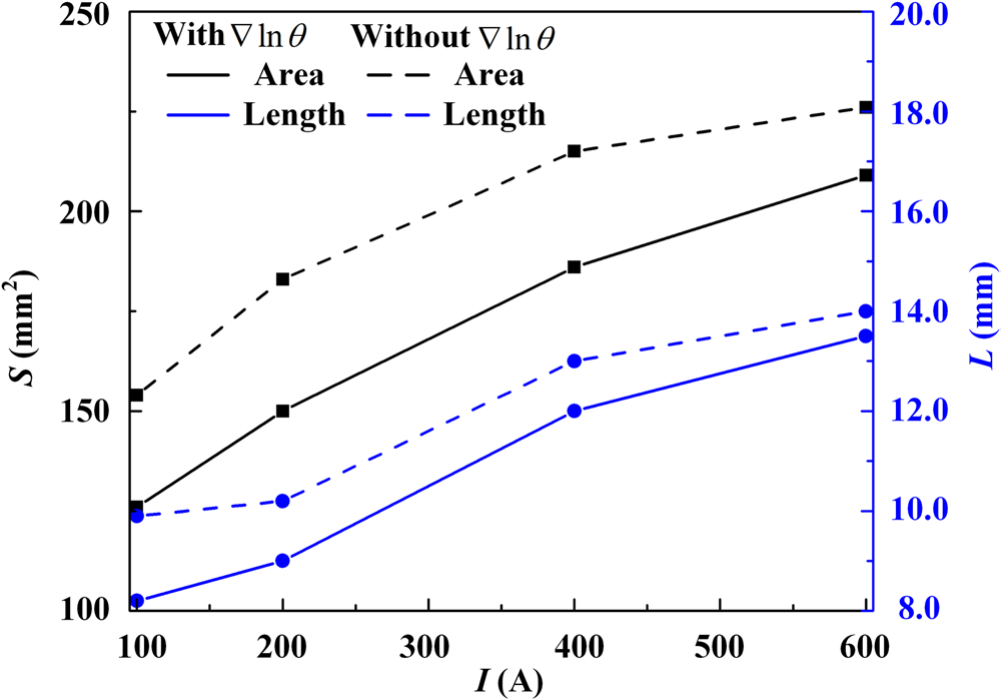
Variations of the area and maximum axial width of the transition region for the heavy-particle subsystem with the arc currents (solid lines), also compared with the modeling results (dashed lines) with no consideration of the terms containing $\vec{{\rm{\nabla }}}\,{\rm{l}}{\rm{n}}\,\theta$ in Equations (10) and (11).

Secondly, for an NLTE plasma system, because there exist spatial temperature distributions of electrons and heavy particles, only two of the three parameters *T*_e_, *T*_h_ and *θ* are independent; that is to say, the spatial gradient of *θ* has to be considered in the non-equilibrium model. This has been embodied in the species perturbation functions with the terms containing $\vec{{\rm{\nabla }}}\,{\rm{l}}{\rm{n}}\,\theta$ [Equations (4) and (5)], and also appears in the energy flux terms of the electron and heavy-particle energy conservation equations, i.e., $\vec{\nabla }\cdot ({\lambda }_{{\rm{e}}}^{{\rm{\theta }}}{T}_{{\rm{e}}}\vec{\nabla }\,\mathrm{ln}\,\theta )$ in Equation (10) and $\overrightarrow{\nabla }\cdot [({\lambda }_{{\rm{i}}}^{{\rm{\theta }}}+{\lambda }_{{\rm{a}}}^{{\rm{\theta }}}){T}_{{\rm{e}}}\overrightarrow{\nabla }\,\mathrm{ln}\,\theta ]$ in Equation (11). These energy flux terms represent the mutual interactions between the subsystems of electrons and heavy particles, physically based on the assumption that there exits strong coupling between electrons and heavy particles through the work and particle transfer between these two subsystems due to the occurrence of the chemical reactions and diffusions in our model^23,24^, instead of regarding them as two isolated systems in the previous simplified models^27,28^. Therefore, from the aspect of energy transfer, the electrons not only “feel” the existence of heavy particles through the direct collisions with them, but also “feel” the non-uniform distribution of the heavy particles due to the unbalanced diffusion driving force; and vice versa. It is anticipated that this energy exchange process related to the spatial gradient of the temperature ratio would become dominated in the region with a high gradient of *θ*, as illustrated in Region III of Fig. 3. And it would weaken the significant difference between *T*_e_ and *T*_h_. By comparing the modeling results in Fig. 4(a,b), we can see clearly that: (i) The predicted transition region becomes larger if the terms $\overrightarrow{\nabla }\cdot ({\lambda }_{{\rm{e}}}^{{\rm{\theta }}}{T}_{{\rm{e}}}\overrightarrow{\nabla }\,\mathrm{ln}\,\theta )$ and $\overrightarrow{\nabla }\cdot [({\lambda }_{{\rm{i}}}^{{\rm{\theta }}}+{\lambda }_{{\rm{a}}}^{{\rm{\theta }}}){T}_{{\rm{e}}}\overrightarrow{\nabla }\,\mathrm{ln}\,\theta ]$ are not included in Equations (10) and (11). And this discrepancy becomes much more significant with the increase of the arc current from 100 A to 600 A, as shown in Fig. 5. Therefore, we can conclude that the energy transfer terms $\overrightarrow{\nabla }\cdot ({\lambda }_{{\rm{e}}}^{{\rm{\theta }}}{T}_{{\rm{e}}}\overrightarrow{\nabla }\,\mathrm{ln}\,\theta )$ and $\overrightarrow{\nabla }\cdot [({\lambda }_{{\rm{i}}}^{{\rm{\theta }}}+{\lambda }_{{\rm{a}}}^{{\rm{\theta }}}){T}_{{\rm{e}}}\overrightarrow{\nabla }\,\mathrm{ln}\,\theta ]$ in Equations (10) and (11) can be regarded as a negative feedback control of the temperature difference between electrons and heavy particles, together with the elastic collision energy transfer process, ${Q}_{{\rm{eh}}}^{{\rm{el}}}$, and the inelastic energy transfer related to the chemical reactions, ${Q}_{{\rm{e}}}^{{\rm{inel}}}$, to drive the non-equilibrium plasma system to an equilibrium one. (ii) Corresponding to the enlarge of the predicted transition region for the case in Fig. 4(b), the spatial distribution of the current density becomes flatter along the radial direction with a lower maximum current density at the arc axis compared with their counterparts using the complete non-equilibrium model in Fig. 4(a). This also leads to a significant discrepancy of the calculated distributions of the electron and heavy-particle temperatures in the equilibrium region close to the arc axis between these two cases.

## Methods and Validation

For a typical atmospheric free-burning argon arc as shown in Fig. 1(a), the calculation domain (ABCDEOFGA) employed in this study is illustrated in Fig. 1(b) with the geometrical dimensions. The plasma generator is composed of a cylindrical tungsten cathode and a flat copper anode operating at specified arc currents. The diameter and length of the tungsten cathode are 4.0 and 15.0 mm, respectively, with a tip angle of 60°, while the thickness and radius of the copper anode are 2.0 mm and 25.0 mm, respectively. The electrode gap spacing between the cathode tip and the anode surface is fixed at 8.0 mm. As shown in Fig. 1(b), the whole calculation domain can be divided into five regions, i.e., the two solid electrode bodies, two electrode sheaths and one arc column region. Since the collisionless sheath near electrodes departures from the quasi-charge neutrality condition with its thickness typically on the magnitude of 1.0 μm at atmospheric pressure^29^ which is much smaller than that of the arc column, no spatial cell is set for these two sheaths. The sheath serves as an interface between the plasma bulk and the electrodes. In this study, the physical-mathematical model for a free-burning argon arc in an axisymmetric coordinate (*r*, *z*) includes three sub-models, i.e., a two-dimensional (2-D) heat and electric conduction model for the solid cathode and anode bodies, a one-dimensional (1-D) collisionless electrode sheath model, and a 2-D NLTE-NLCE arc column model. The non-equilibrium model for the plasma bulk is based on our previous research which includes the governing equations and the self-consistent transport properties with the consideration of three components, i.e., argon atoms, firstly-ionized ions, and electrons. The governing equations with the necessary transport properties of the non-equilibrium argon plasmas^23,24^ are listed as follows:

8 $$\overrightarrow{\nabla }\cdot ({n}_{{\rm{j}}}{\overrightarrow{v}}_{0})=-\overrightarrow{\nabla }\cdot ({n}_{{\rm{j}}}{\overrightarrow{V}}_{{\rm{j}}})+{\dot{n}}_{{\rm{j}}},$$

9 $$\overrightarrow{\nabla }\cdot (\rho {\overrightarrow{v}}_{0}{\overrightarrow{v}}_{0})=-\overrightarrow{\nabla }p+\overrightarrow{\nabla }\cdot \overleftrightarrow{\tau }+\overrightarrow{j}\times \overrightarrow{B},$$

10 $$\vec{{\rm{\nabla }}}\cdot [\frac{5}{2}{k}_{{\rm{B}}}{n}_{{\rm{e}}}{T}_{{\rm{e}}}({\vec{v}}_{0}+{\vec{V}}_{{\rm{e}}})]=\vec{{\rm{\nabla }}}\cdot ({\lambda }_{{\rm{e}}}\vec{{\rm{\nabla }}}{T}_{{\rm{e}}})+\vec{{\rm{\nabla }}}\cdot ({\lambda }_{{\rm{e}}}^{\theta }{T}_{{\rm{e}}}\vec{{\rm{\nabla }}}\,{\rm{l}}{\rm{n}}\,\theta )-e{n}_{{\rm{e}}}{\vec{V}}_{{\rm{e}}}\cdot \vec{E}-{Q}_{{\rm{e}}{\rm{h}}}^{{\rm{e}}{\rm{l}}}+{Q}_{{\rm{e}}}^{{\rm{i}}{\rm{n}}{\rm{e}}{\rm{l}}}-{U}_{{\rm{R}}},$$

11 $$\vec{{\rm{\nabla }}}\cdot \{\frac{5}{2}{k}_{{\rm{B}}}{T}_{{\rm{h}}}[{n}_{{\rm{a}}}({\vec{v}}_{0}+{\vec{V}}_{{\rm{a}}})+{n}_{{\rm{i}}}({\vec{v}}_{0}+{\vec{V}}_{{\rm{i}}})]\}=\vec{{\rm{\nabla }}}\cdot ({\lambda }_{{\rm{h}}}\vec{{\rm{\nabla }}}{T}_{{\rm{h}}})+\vec{{\rm{\nabla }}}\cdot [({\lambda }_{{\rm{i}}}^{\theta }+{\lambda }_{{\rm{a}}}^{\theta }){T}_{{\rm{e}}}\vec{{\rm{\nabla }}}\,{\rm{l}}{\rm{n}}\,\theta ]+e{n}_{{\rm{i}}}{\vec{V}}_{{\rm{i}}}\cdot \vec{E}+{Q}_{{\rm{e}}{\rm{h}}}^{{\rm{e}}{\rm{l}}}+{Q}_{{\rm{h}}}^{{\rm{i}}{\rm{n}}{\rm{e}}{\rm{l}}},$$

12 $$\overrightarrow{\nabla }\cdot (\sigma \overrightarrow{\nabla }\phi )=0,$$

where the subscript “j” indicates the different species in plasmas including electrons and heavy particles with argon ions and atoms which are represented by the subscripts “e”, “h”, “i” and “a”, respectively; ${Q}_{{\rm{eh}}}^{{\rm{el}}}$ represents the energy exchange of the elastic collisions between electrons and heavy particles, while the terms ${Q}_{{\rm{e}}}^{{\rm{inel}}}$ and ${Q}_{{\rm{h}}}^{{\rm{inel}}}$ stand for the energy exchanges related to the chemical reactions for the electron and heavy-particle subsystems, respectively. The physical meanings of other terms are listed in refs^23,24^. The boundary conditions for the modelling results in Figs 2~5 are listed in Table 1.

**Table 1:** Boundary conditions for the modelling results in Figs 2~5.

	OF	FG	GA	AB	BC	CD	DE	EO
*v*_z_ (m/s)	—	$\frac{\partial {v}_{{\rm{z}}}}{\partial r}=0$	—	—	—	$\frac{\partial {v}_{{\rm{z}}}}{\partial r}=0$	$\frac{\partial (\rho {v}_{{\rm{z}}})}{\partial z}=0$	—
*v*_r_ (m/s)	—	0	—	—	—	$\frac{\partial (\rho r{v}_{{\rm{r}}})}{\partial r}=0$	$\frac{\partial {v}_{{\rm{r}}}}{\partial z}=0$	—
*n*_e_ (m^−3^)	—	$\frac{\partial {n}_{{\rm{e}}}}{\partial r}=0$	—	—	—	$$\begin{array}{c}\frac{\partial {n}_{{\rm{e}}}}{\partial r}=0,{v}_{{\rm{r}}} > 0\\ 0,{v}_{{\rm{r}}}\le 0\end{array}$$	$$\begin{array}{c}\frac{\partial {n}_{{\rm{e}}}}{\partial z}=0,{v}_{{\rm{z}}} < 0\\ 0,{v}_{{\rm{z}}}\ge 0\end{array}$$	—
*n*_a_ (m^-3^)	—	$\frac{\partial {n}_{{\rm{a}}}}{\partial r}=0$	—	—	—	$$\begin{array}{c}\frac{\partial {n}_{{\rm{a}}}}{\partial r}=0,{v}_{{\rm{r}}} > 0\\ \frac{p}{{k}_{{\rm{B}}}{T}_{{\rm{h}}}},{v}_{{\rm{r}}}\le 0\end{array}$$	$$\begin{array}{c}\frac{\partial {n}_{{\rm{a}}}}{\partial z}=0,{v}_{{\rm{z}}} < 0\\ \frac{p}{{k}_{{\rm{B}}}{T}_{{\rm{h}}}},{v}_{{\rm{z}}}\ge 0\end{array}$$	—
*T*_h_ (K)	$\frac{\partial {T}_{{\rm{h}}}}{\partial r}=0$	$\frac{\partial {T}_{{\rm{h}}}}{\partial r}=0$	$\frac{\partial {T}_{{\rm{h}}}}{\partial r}=0$	500	$\frac{\partial {T}_{{\rm{h}}}}{\partial r}=0$	$$\begin{array}{c}\frac{\partial {T}_{{\rm{h}}}}{\partial r}=0,{v}_{{\rm{r}}} > 0\\ 1000,{v}_{{\rm{r}}}\le 0\end{array}$$	$$\begin{array}{c}\frac{\partial {T}_{{\rm{h}}}}{\partial z}=0,{v}_{{\rm{z}}} < 0\\ 1000,{v}_{{\rm{z}}}\ge 0\end{array}$$	500
*T*_e_(K)	—	$\frac{\partial {T}_{{\rm{e}}}}{\partial r}=0$	—	—	—	$$\begin{array}{c}\frac{\partial {T}_{{\rm{e}}}}{\partial r}=0,{v}_{{\rm{r}}} > 0\\ 1000,{v}_{{\rm{r}}}\le 0\end{array}$$	$$\begin{array}{c}\frac{\partial {T}_{{\rm{e}}}}{\partial z}=0,{v}_{{\rm{z}}} < 0\\ 1000,{v}_{{\rm{z}}}\ge 0\end{array}$$	—
*ϕ*(V)	$\frac{\partial \varphi }{\partial r}=0$	$\frac{\partial \varphi }{\partial r}=0$	$\frac{\partial \varphi }{\partial r}=0$	0	$\frac{\partial \varphi }{\partial r}=0$	$\frac{\partial \varphi }{\partial r}=0$	$\frac{\partial \varphi }{\partial z}=0$	$-\sigma \frac{\partial \varphi }{\partial z}=\frac{I}{S}$

^*^The symbols *v*_z_ and *v*_r_ represent the axial and radial components of the velocity vector in the (*r*, *z*) coordinate; while *I* and *S* stand for the arc current and the area of the rear surface of the cathode, respectively.

Since we focus on the non-equilibrium effects in the arc column region, a simple collisionless ion-electron sheath model^30^ is employed in this report. In this simplified sheath model, the electrode sheath works as an information exchange interface between the solid electrode and the arc column. It gives the relationship between the sheath voltage drop and the current density; provides the potential and heat flux to the plasma side; provides the current density and heat flux to the electrodes based on the solution of the conservation equations of the sheath. That is to say, the cathode and anode sheaths are coupled in the form of the boundary conditions with the other parts of the arc system. The preceding complete non-equilibrium plasma model is solved numerically using a computer code based on the SIMPLE-like algorithm^31^ to study the NLTE-NLCE synergistic effects on the energy exchange and particle balance processes in an atmospheric free-burning argon arc working as a typical AP-LTP model.

**Figure 6:**
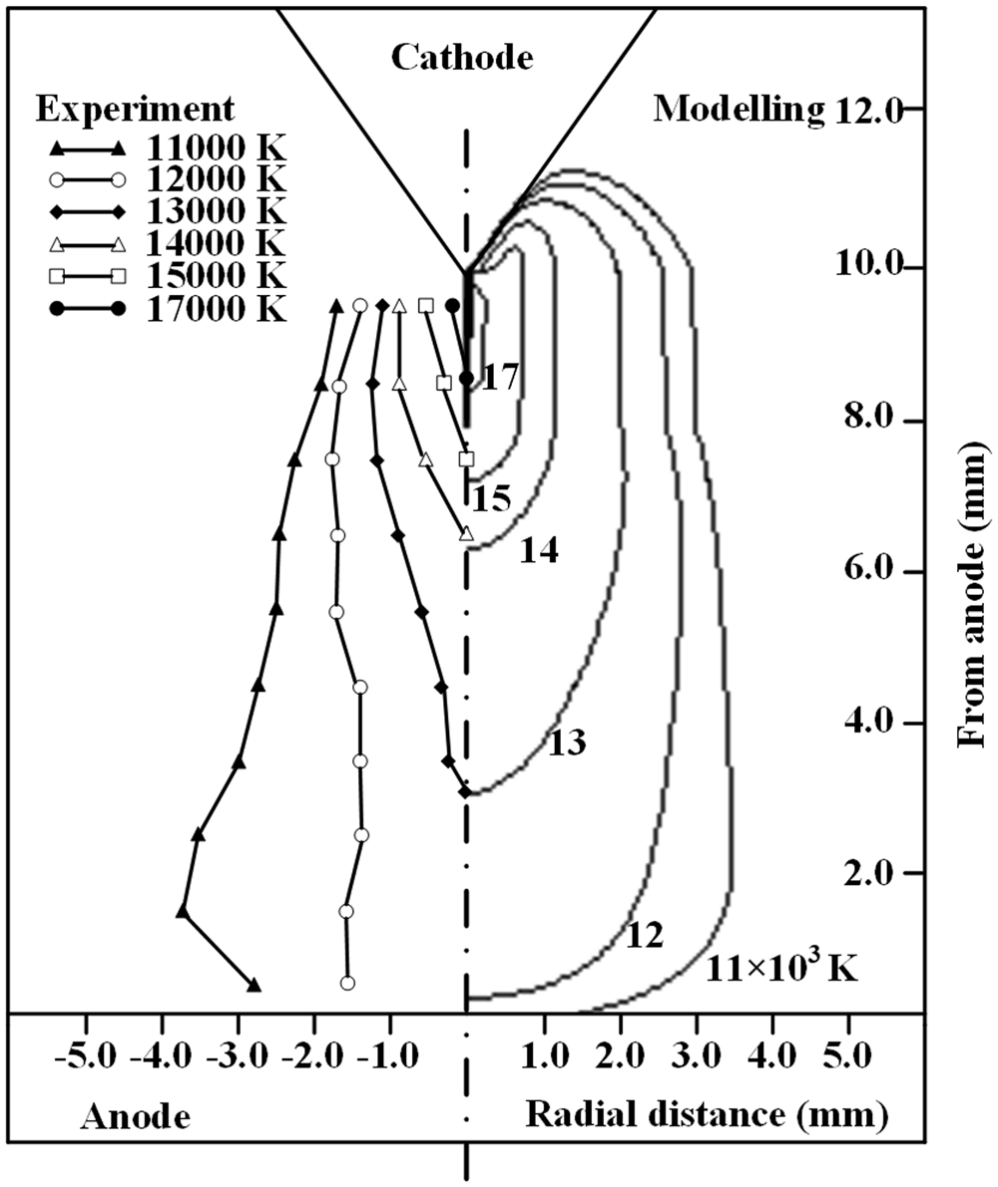
Comparison of isotherms of the calculated electron temperature (right) with the measured results^32^ (left) of an atmospheric free-burning argon arc for the case of *I* = 100 A and *d* = 10 mm.

**Figure 7:**
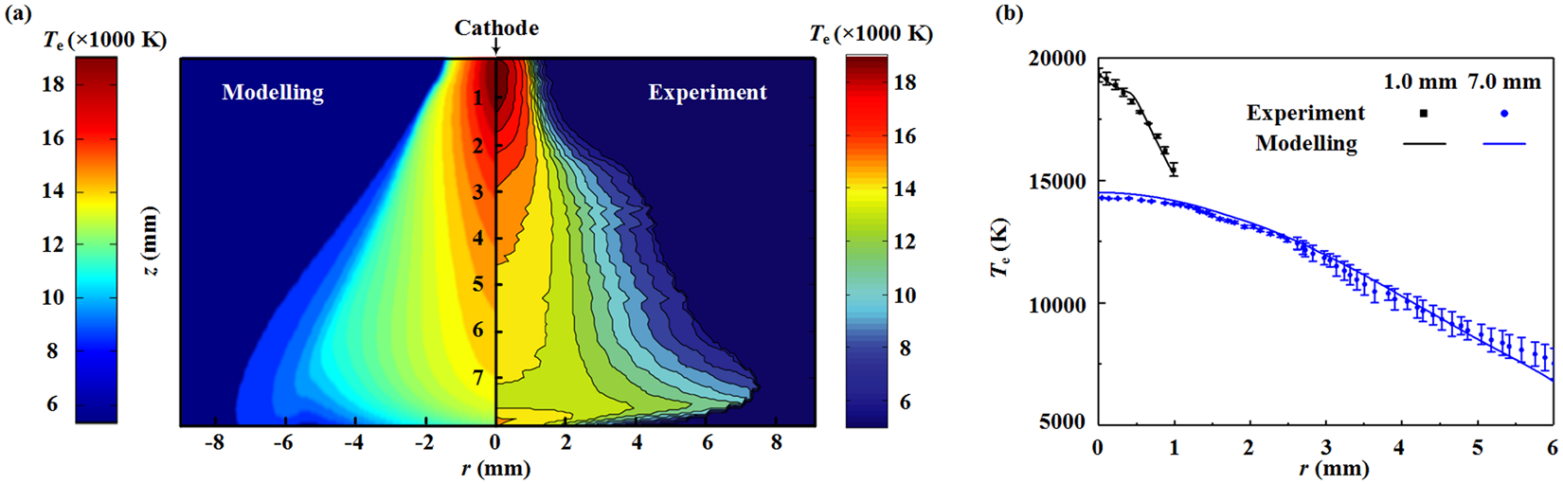
Comparisons of the calculated and measured^34^ 2-D electron temperature distributions (**a**), and the radial profiles of *T*_e_ at *L* = 1.0 and 7.0 mm (**b**) for a TIG argon arc (*I* = 200 A, *d* = 8.0, *Q* = 12.0 slpm).

In previous studies, many experimental investigations on the thermal and electrical characteristics of arc plasmas have been conducted, e.g., the work presented in^32–35^. In this section, we have done two validations (one is the free-burning argon arc, another is the tungsten inert gas (TIG) welding arc) before using this novel complete non-equilibrium model to study the NLTE-NLCE synergistic effects on the non-equilibrium characteristics of the AP-LTPs. A comparison between the experimentally measured temperature distributions of a free-burning argon arc based on the optical emission spectroscopy measurements^32^ and the calculated electron temperatures for the arc current of *I* = 100 A and the electrode gap spacing of *d* = 10 mm is shown in Fig. 6. It is seen that the calculated spatial distribution of *T*_e_ is consistent well with that of the measured data, especially for the temperature profile along the arc axis. The calculated total discharge voltage including the potential drops through the arc column and the electrode sheaths is 23.2 V, which is also very close to the measured value of 22 V, with a relative discrepancy of 5.1%. For the TIG argon arcs, the comparisons of the calculated and measured 2-D electron temperature distributions, as well as the radial profiles of *T*_e_ at the axial locations of *L* = 1.0 and 7.0 mm downstream of the cathode tip are presented in Fig. 7(a,b), respectively, under the operating conditions of *I* = 200 A, *d* = 8.0 mm and a gas inlet flowrate of *Q* = 12.0 slpm along the cathode surface^34^. The maximum values of *T*_e_ from modeling and measurements are both around 19000 K at *L* = 1.0 mm, which is also qualitatively consistent with the measured data in ref.^35^. The maximum discrepancies between the calculated and measured values of *T*_e_ at *L* = 1.0 and 7.0 mm are 1.7% and 1.5%, respectively. The preceding comparisons indicate the reliability of the non-equilibrium physical-mathematical model, as well as the developed computer codes in this report.
